# A Flexible Temperature Sensor for Noncontact Human-Machine Interaction

**DOI:** 10.3390/ma14237112

**Published:** 2021-11-23

**Authors:** Shiqi Chen, Xiaolong Han, Peng Hong, Yue Zhang, Xiangyu Yin, Bingwei He

**Affiliations:** 1 College of Mechanical Engineering and Automation, Fuzhou University, Fuzhou 350108, China; 210220045@fzu.edu.cn (S.C.); 210227080@fzu.edu.cn (P.H.); bw_he@aliyun.com (B.H.); 2 Maynooth International Engineering College, Fuzhou University, Fuzhou 350108, China; 831902106@fzu.edu; 3 Fujian Engineering Research Center of Joint Intelligent Medical Engineering, Fuzhou 350108, China; 4 College of Chemical Engineering, Fuzhou University, Fuzhou 350108, China

**Keywords:** temperature-sensitive, noncontact sensing, flexible sensor

## Abstract

Flexible sensors have attracted extensive attention because of their promising applications in the fields of health monitoring, intelligent robots, and electronic skin, etc. During the COVID-19 epidemic, noncontact control of public equipment such as elevators, game consoles, and doors has become particularly important, as it can effectively reduce the risk of cross-infection. In this work, a noncontact flexible temperature sensor is prepared via a simple dip-drying progress, in which poly(3,4-ethylenedioxythiophene):poly(4-styrene sulfonate) (PEDOT:PSS) and printer paper served as the sensing material and the flexible substrate, respectively. We combined the highly sensitive temperature-responsive property of PEDOT:PSS with the good hygroscopicity of printer paper. The prepared sensor shows high sensitivity and good stability in noncontact sensing mode within the temperature range of 20–50 °C. To prove the practicability of the noncontact temperature sensor, a 3 × 2 sensing array is prepared as a noncontact human-machine interface to realize the interaction between player and “Pound-A-Mole game” and a Bluetooth car. These two demos show the sensor′s ability to perceive nearby temperature changes, verifying its application potential as a noncontact human-machine interaction interface.

## 1. Introduction

With the continuous development of sensing technology, people have shown great demand for advanced flexible electronic devices [1,2]. Among them, flexible temperature sensors have attracted the attention of many specialists and scholars domestically and abroad in recent years due to their unique properties of high efficiency, seamless contact with the dynamic human body, continuity, and longterm temperature monitoring [3,4,5]. When heat transfer occurs between the human body and its surrounding things, thermometers with high precision and continuous temperature measurement are often used to monitor the interaction between humans and machines or between humans and the environment. In the field of intelligent medical care, researchers often monitor the patient′s body temperature and other physiological parameters to diagnose various diseases [6,7,8]. In addition, real-time temperature response shows great application prospects in fields such as artificial electronic skin and emotion recognition [9,10,11,12]. Thus, it can be said that temperature as a basic physical parameter, as well as being used to monitor physiological activities and health, can be used to monitor and control human-machine interaction [13,14].

Flexible temperature sensors often adopt multi-point ways to continuously measure the surface temperature of irregular objects [15], which requires the sensors to have the following characteristics: (1) The temperature sensing performance in terms of measurement accuracy, sensitivity, response time, and repeatability should be matched with traditional thermometers. (2) The flexible sensor should be highly sensitive and have longterm stability in a temperature range of 20 °C~50 °C. (3) Facile fabrication on a large scale is also very important for the sensors’ application, because multipixel sensing arrays are often integrated to achieve simultaneous measurement of multipoint temperature. (4) Mechanical robustness and anti-interference are also essential for flexible temperature sensors. In practical applications, flexible temperature sensors are often used for dynamic surface temperature measurement. During the measurement, the sensor will be twisted and deformed with the surface, so good mechanical robustness is required to ensure its longterm use [8]. Moreover, the sensitive electrodes of flexible temperature sensors are mostly made of graphene [16], carbon nanotubes [17], and metal nanomaterials [18,19], which are not only highly sensitive to temperature, but also extremely sensitive to strain and pressure, which will cause serious signal crosstalk and make it difficult to distinguish whether the electrical signal changes are determined by temperature or deformation. Therefore, anti-interference is very important to ensure the accuracy of temperature measurement.

Poly(3,4-ethylenedioxythiophene):poly(4-styrene sulfonate) (PEDOT:PSS), an aqueous solution of PEDOT and PSS, has attracted more and more attentions due to its high optical transparency and adequate conductivity (higher than 1 S cm^−1^). With the fabrication techniques of inkjet printing [20], direct-print dispensing [21], and dip-drying [22], PEDOT:PSS films can be obtained and be subsequently integrated into flexible electronic devices. In these flexible electronic devices, the PEDOT:PSS films usually serve as active components, because they are sensitive to some environmental factors, such as humidity, pH, and temperature. The resistance of the PEDOT:PSS films will decrease with temperature increasing, humidity decreasing, and pH increasing. Although many flexible electronics were developed based on PEDOT:PSS, little research has been conducted on their ability as a non-contact interface to control human-machine interaction. In addition, a flat PEDOT:PSS film is usually insensitive to strain and pressure, unless the PEDOT:PSS is prepared on a substrate with good elasticity or special microstructures. Based on these feature, we prepared a flexible temperature-sensitive paper by dipping common flexible printer paper in PEDOT:PSS solution and then drying it. Due to the high sensitivity of the PEDOT:PSS paper to temperature (−21.5 × 10^−3^ °C^−1^ in temperature range of 20–50 °C), the temperature-sensitive paper can quickly detect the proximity of objects with temperature differences, enabling it to identify the human proximity and act as a control interface to achieve noncontact human-machine interaction.

## 2. Experiment and Characterization

### 2.1. Materials

White printer papers with standard quality of 60 g m^−2^ and 80 g m^−2^, and polyethylene terephthalate (PET) film with a thickness of 0.125 mm were used as flexible substrates after degreasing with acetone, dewatering with ethanol. PEDOT:PSS (1.5 wt.%) and dimethyl sulfoxide (DMSO) were used as received (Aladdin, Shanghai, China). Deionized water (18.2 MΩ cm^−1^) from a Milli-Q system (Human Power I Plus, Korea) was used to prepare all aqueous solutions. Electrical contacts were made using silver paste (SCP003, Electrolube, Leicestershire, UK).

### 2.2. Preparation of PEDOT:PSS Temperature Sensor

**Figure 1:**
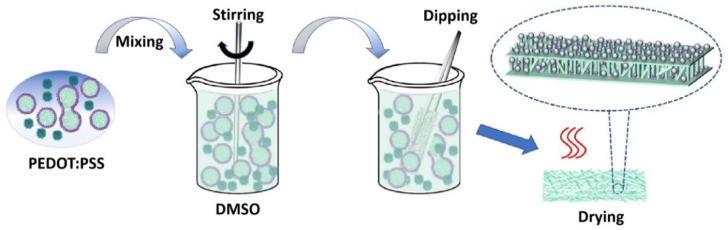
Schematic diagram of the preparation process for flexible temperature sensors.

The noncontact temperature-sensitive paper was prepared via an easy-to-operate dip-drying progress, in which the printer papers or PET film acted as the flexible substrate, and the PEDOT:PSS acted as temperature-sensitive material. The schematic diagram of the preparation process is shown in Figure 1. Specifically, PEDOT:PSS solution and deionized water with a mass ratio of 2:1 was mixed, then 5.0 wt.% DMSO was added under magnetic stirring at 600 rpm for 10 min to obtain a uniformly dispersed homogeneous solution. At the same time, the preprocessed printer papers and PET film were cut into a size of 10 mm × 20 mm, and one side of the printer papers was encapsulated with transparent adhesive tape, so that the solution could only be deposited on one side of the printer papers. The flexible substrates were vertically immersed in the above PEDOT:PSS solution for 90 s and then heated at 100 °C for 30 min. Finally, temperature sensors were obtained by using conductive silver paste to connect the temperature-sensitive papers/PET with external circuit. Except for special instructions, all experiments were carried out at 28 ± 1 °C of temperature and 70 ± 1% of relative humidity.

### 2.3. Characterization

The optical morphology of the commercial printer papers and the fabricated temperature-sensitive papers were characterized using an Optical Microscope (DM750, Leica, Weztlar, Germany). A 3D optical surface profiler was used to map the surface topography and determine surface roughness of the specimens. The cross-sectional morphology of the sensors was determined by FE-SEM tests (Nova NanoSEM 230, FEI, MA, USA). The temperature-sensitive test system includes a DZF-6050 vacuum drying oven (Shanghai Jinghong Experimental Equipment Co., Ltd., Shanghai, China), a DMM6500 digital acquisition system multimeter (KEITHLEY, Cleveland, OH, USA) and a thermocouple thermometer instrument (YET-601, Yuwexa, Guangdong, China). In order to more accurately measure the temperature of the sensor, a thermocouple probe was pasted to the lower surface of the flexible temperature sensors. Then the flexible temperature sensors were placed into a preheated oven and connected to a multimeter (DMM6500, KEITHLEY, OH, USA) to monitor the resistance change. As the temperature rose, the resistance of the sensors was recorded in real-time using a two-wire resistance measurement mode. The measurement frequency was 1 kHz, and the tested temperature ranged from 20 to 80 °C. All tests were performed at least five times, and the average value is reported here.

## 3. Results and Discussion

### 3.1. Preparation and Characterization

**Figure 2:**
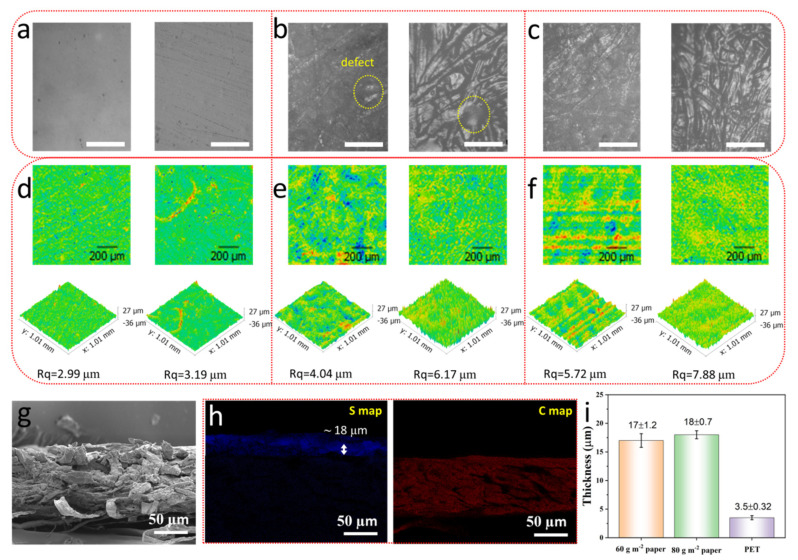
Morphology of PEDOT:PSS film on different flexible substrates. Optical morphology of PEDOT:PSS film prepared on (**a**) PET film, (**b**) 60 g m^−2^ printer paper, and (**c**) 80 g m^−2^ printer paper. Scale bar = 100 µm. 3D profile of PEDOT:PSS film prepared on (**d**) PET film, (**e**) 60 g m^−2^ printer paper, and (**f**) 80 g m^−2^ printer paper. The left and right images in each figure correspond to the morphology of the initial substrate and the substrate after depositing PEDOT:PSS, Rq is the root mean square roughness. (**g**) Cross-sectional SEM morphology of the 80 g m^−2^ PEDOT:PSS paper and (**h**) its EDS mapping for elements of S and C; (**i**) Thickness of PEDOT:PSS layer on different substrates.

The temperature-sensitive paper was prepared by a simple dip-drying progress as shown in Figure 1. To study the effect of flexible substrates on the temperature-sensitive property, printer papers with different quality (60 g m^−2^ and 80 g m^−2^) and PET film with a thickness of 125 µm were dipped into PEDOT:PSS solution for 90 s, respectively. The characteristics of the PEDOT:PSS film formed on different flexible substrates were observed in detail using an optical microscope. Obviously, the surface of the PET film before and after dipping was very smooth, indicating that the PEDOT:PSS uniformly coats on the PET surface after dipping (Figure 2a). In contrast, the fiber structure of the two paper-based flexible substrates was clearly visible, and the 60 g m^−2^ printer paper had more substantial voids in the fibrous material than the 80 g m^−2^ printer paper, as shown in Figure 2b,c. After dipping in PEDOT:PSS solution for 90 s, PEDOT:PSS was deposited on and between paper fibers, but it could not make up for the large-size defects of 60 g m^−2^ printer paper, so incomplete and complete films were formed on the 60 g m^−2^ and 80 g m^−2^ printer papers, respectively. To further judge the deposition of PEDOT:PSS on the three flexible substrates, we conducted a 3D profile observations on the three substrates before and after dipping in PEDOT:PSS solution for 90 s. Figure 2d–f shows that compared with the initial state, the thickness of the three flexible substrates increased after dipping, which indicates that PEDOT:PSS successfully deposited on the flexible substrates. Moreover, the more significant change of the paper substrates in thickness and the root mean square roughness (Rq) proves more PEDOT:PSS deposition. In addition, Figure 2e,f shows that the initial papers with different qualities have many defects, and the 60 g m^−2^ printer paper is more serious. After depositing PEDOT:PSS, the number and size of the paper defects were significantly reduced, further indicating that a complete conductive film was formed on the paper substrates, which is one of the decisive factors for obtaining uniform temperature-sensitive performance. To future determine the thickness of the PEDOT:PSS film deposited on different substrates, cross-sectional SEM morphology (Figure 2g) and the EDS mapping for elements of S and C (Figure 2h) were carried out. The thickness of the PEDOT:PSS film was 17 ± 1.2 µm on 60 g m^−2^ printer paper, 18 ± 0.7 µm on 80 g m^−2^ printer paper, and 3.5 ± 0.3.2 µm on FET film.

**Figure 3:**
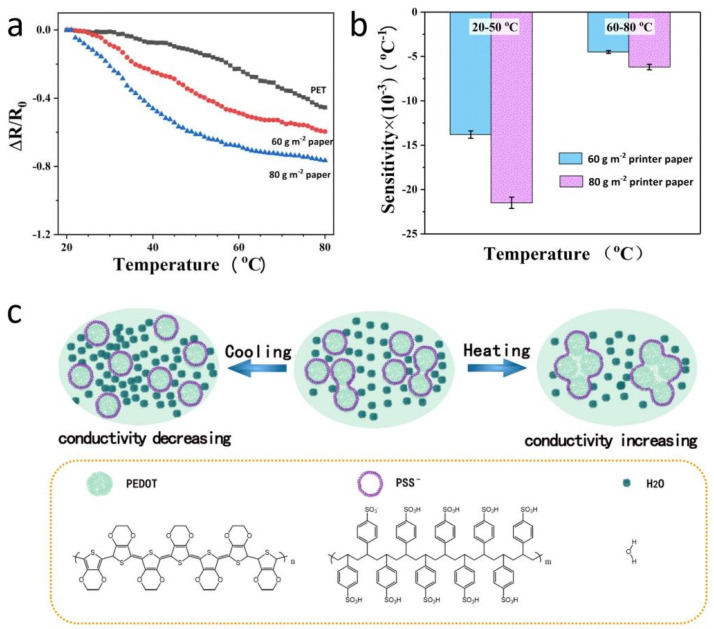
Effect of flexible substrates on sensitivity. (**a**) Variation curves of the normalized resistance (ΔR/R_0_) with respect to temperature for three flexible temperature sensors; (**b**) the sensitivity of paper-based sensors within different temperature ranges; (**c**) schematic diagram of temperature response mechanism for PEDOT:PSS based sensor.

Figure 3a shows the variation curves of normalized resistance (ΔR/R_0_) with respect to temperature for three flexible temperature sensors (the initial resistivity of the 60 g m^−2^, 80 g m^−2^ temperature-sensitive papers and the temperature-sensitive PET are 22.2, 15.1, and 18.9 mΩ·cm, respectively, comparable to the value reported in the literature [23,24]). From the curves, the sensitivity of each sensor (Figure 3b) can be calculated according to the following calculation: S = ΔR/(R_0_·ΔT), where R_0_ is the initial resistance, ΔR is the resistance change, and ΔT is the temperature change. It can be seen from Figure 3b that within the temperature range of 20–80 °C, the sensitivity of the three temperature sensors based on different flexible substrates is sorted as follows: 80 g m^−2^ printer paper > 60 g m^−2^ printer paper > PET film. Obviously, the sensitivity of PET-based sensors is the smallest. In the temperature range of 30–80 °C, its sensitivity is −9.3 × 10^−3^ °C^−1^, but in 20–30 °C, its temperature sensitivity is only −1.1 × 10^−3^ °C^−1^, which determines that it cannot be used as a flexible interface to achieve human-machine interaction. At the same time, the sensitivity of the two paper-based sensors showed a higher temperature sensitivity in the range of 20–50 °C (−21.5 × 10^−3^ °C^−1^ for 80 g m^−2^ printer paper, and −13.8 × 10^−3^ °C ^−1^ for 60 g m^−2^ printer paper), and a relatively low sensitivity within the temperature range of 50–80 °C (Figure 3b). Obviously, the sensitivity of 80 g m^−2^ printer paper was still better than 60 g m^−2^ (−6.2 × 10^−3^ °C^−1^ for 80 g m^−2^ printer paper, and −4.5 × 10^−3^ °C^−1^ for 60 g m^−2^ printer paper) in the high temperature range, so the 80 g m^−2^ printer paper was selected as the flexible substrate to prepare high-performance temperature sensor.

It is known that PEDOT is a black powder with poor water solubility, which is of little use in practical applications. After adding PSS, we could obtain conductive polyelectrolyte complex with improved solubility in aqueous solution. The conductivity of the complex depends on the network characteristics of PEDOT: PSS [25,26,27] and intermolecular water content (Figure 3c). Increasing the intermolecular water content will reduce the adhesion between the molecules in the PEDOT:PSS network, thereby reducing the conductivity. On the contrary, decreasing the intermolecular water content will increase the conductivity. Heating will inevitably decrease the intermolecular water content in the PEDOT:PSS network, and cooling will play an opposite effect, so the PEDOT:PSS film exhibits temperature-sensitive characteristics. In addition, the high sensitivity of the paper-based sensors compared to the PET-based sensor is also determined by the good moisture absorption of the paper substrates themselves, while the temperature sensitivity of 80 g m^−2^ printer paper is better than that of 60 g m^−2^ printer paper because it has more PEDOT:PSS per unit area. Besides, the denser fibrous structure of the 80 g m^−2^ printer paper plays a more obvious role in absorbing and releasing environmental water molecules. Therefore, in the temperature range of 20–50 °C, the temperature-sensitive property of the 80 g m^−2^ printer paper-based sensor is the best, while PET-based sensor is the worst. In addition, for the paper-based sensors, the influence of temperature over 50 °C is almost negligible on the intermolecular water content in the PEDOT:PSS network, and under this condition, the conductivity of the PEDOT:PSS film depends entirely on the hydrogen bonding of the PSS chains. Undoubtedly, high temperature will activate hydrogen bonds, thereby increasing the adhesion between PEDOT molecules and consequently improving the conductivity of the PEDOT:PSS film. Therefore, in the range of 50 to 80 °C, although the sensitivity of 80 g m^−2^ PEDOT:PSS temperature sensitive paper is lower than that in 20–50 °C, its resistance will still decrease as the temperature increases. For PET-based sensor, due to the poor water absorption of the substrate, temperature mainly determines its electrical conductivity by affecting intermolecular hydrogen bonds. Therefore, its sensitivity in the low temperature region (20–30 °C) is low, and the sensitivity in higher temperature region (30–80 °C) is slightly increased.

**Figure 4:**
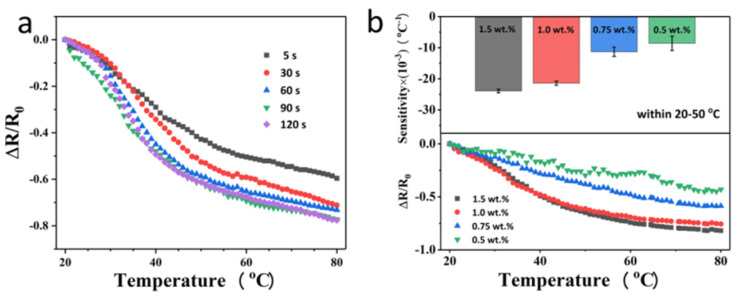
Effect of dipping time and concentration of PEDOT:PSS on the sensitivity of the flexible temperature-sensitive papers. (**a**) Variation of the normalized resistance (ΔR/R_0_) with respect to temperature for sensing papers prepared with different dipping time; (**b**) effect of PEDOT:PSS concentration on the sensing properties. Top: Sensitivity of temperature-sensing papers prepared in 0.5, 0.75, 1.0, and 1.5 wt.% PEDOT:PSS. Bottom: Variation of the normalized resistance (ΔR/R_0_) with respect to temperature for sensing papers prepared in PEDOT:PSS with different concentration. Temperature range: 20–50 °C.

Next, we select the 80 g m^−2^ printer paper-based PEDOT:PSS sensor as the research object to investigate the influence of dipping time and the composition of the PEDOT:PSS solution on its temperature-sensitive performance. Firstly, the printer paper with a size of 10 mm × 20 mm was dipped in the mixture of 1.0 wt.% PEDOT:PSS + 5.0 wt.% DMSO for 5, 30, 60, 90, 120, and 150 s to investigate the effect of dipping time on the performance of the prepared sensors. It can be seen from Figure 4a that with the increase of dipping time, the normalized resistance of the obtained temperature-sensitive papers increases obviously under the same temperature change, proofing its more sensitive temperature-sensing properties. In addition, there is little difference in temperature response of samples dipping for 90 and 120 s. Therefore, we select 90 s as the best dipping time, and on this basis, we further investigate the influence of the concentration of PEDOT:PSS in the mixed solution on the temperature sensitivity of the samples. Figure 4b shows that with the increase of PEDOT:PSS concentration, the sensitivity and stability of the prepared temperature sensor are greatly improved. In the range of 20–50 °C, the sensitivity of temperature-sensitive paper prepared by 1.5 wt.% and 1.0 wt.% PEDOT:PSS is (−23.9 ± 0.57) × 10^−3^ °C^−1^ and (−21.5 ± 0.64) × 10^−3^ °C^−1^, indicating that at this concentrations, PEDOT:PSS can evenly form a film on the printer paper.

### 3.2. Temperature-Sensing Performance

**Figure 5:**
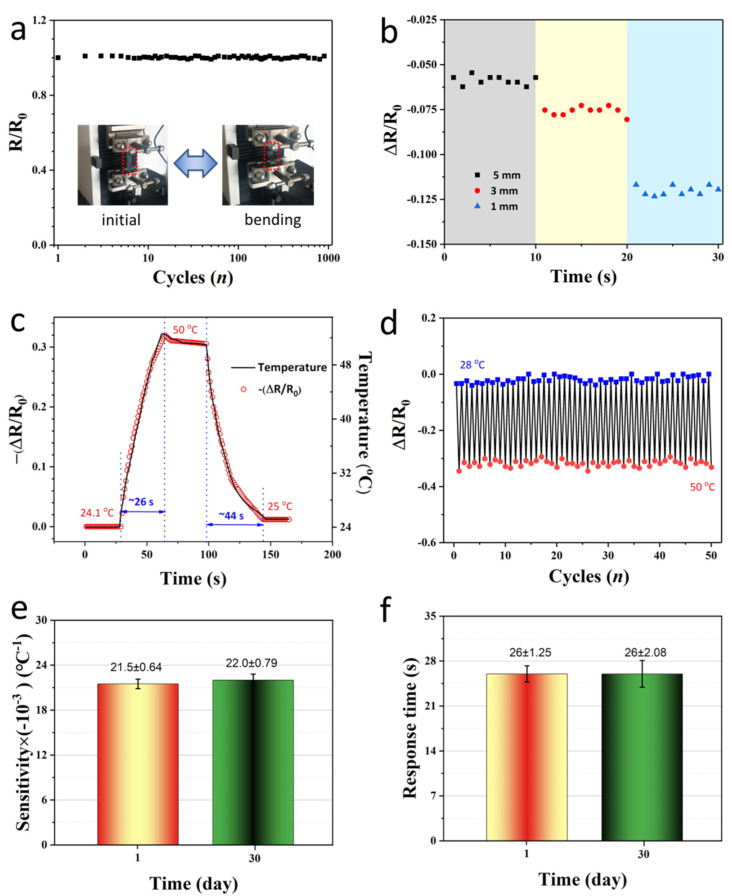
Temperature-sensitive performances. (**a**) Sensing stability of PEDOT:PSS paper buckled from initial to −30% for 1000 times; (**b**) Temperature sensitivity of the PEDOT:PSS paper at a finger proximity distances of 1, 3, and 5 mm, respectively; (**c**) The response time curve ranges from 28 °C to 50 °C; (**d**) Multi-cycles temperature-sensing stability tested between 28 and 50 °C. Comparison of (**e**) sensitivity and (**f**) response time of the same sensor between Day 1 and Day 30.

Based on the above experiments, we prepared flexible temperature-sensing paper by dipping the 80 g m^−2^ printer paper in 1.0 wt.% PEDOT:PSS + 5.0 wt.% DMSO mixed solution for 90 s, and investigated its sensing performance. Figure 5a shows that after the temperature-sensitive paper was repeatedly buckled to 0~−30% at room temperature 1000 times, the resistance of the sample hardly changed, proving its good mechanical stability and practical applicability. Next, we verified its noncontact sensing performance by controlling the distance between the finger and the temperature-sensitive paper at ambient temperature (28 °C). During the tests, the finger approached the sensor paper to a fixed distance of 1, 3, and 5 mm and started recording resistance changes after 10 s. Each distance was maintained for 10 s to investigate the resistance stability of the temperature-sensitive paper. Obviously, the closer the finger is to the temperature-sensitive paper, the more obvious the normalized resistance of the temperature-sensitive paper changes. Even if the distance was 5 mm, the proximity of the finger (~36.5 °C) couold also be detected and kept relatively stable (Figure 5b), which proved the practical application potential of the sensing paper as a noncontact control human-machine interaction interface. Generally, the response time of temperature sensors usually takes several minutes, which is determined by the thermal conductivity of the materials and the air. Figure 5c shows that once the temperature-sensitive paper was placed 5 mm over a heating plate (50 °C), its normalized resistance decreases rapidly. Although the resistance value reaches stable needing about 32 s, the obvious resistance change in a short period of time was sufficient for noncontact human-machine interaction interface. More importantly, the temperature-sensitive paper had stable sensing performance, proven by the good temperature response behavior during repeatedly tested in 28 and 50 °C environment test (Figure 5d), and by the not obvious difference in the sensitivity and response time of the same sensor between Day 1 and Day 30 (Figure 5e,f).

## 4. Applications

**Figure 6:**
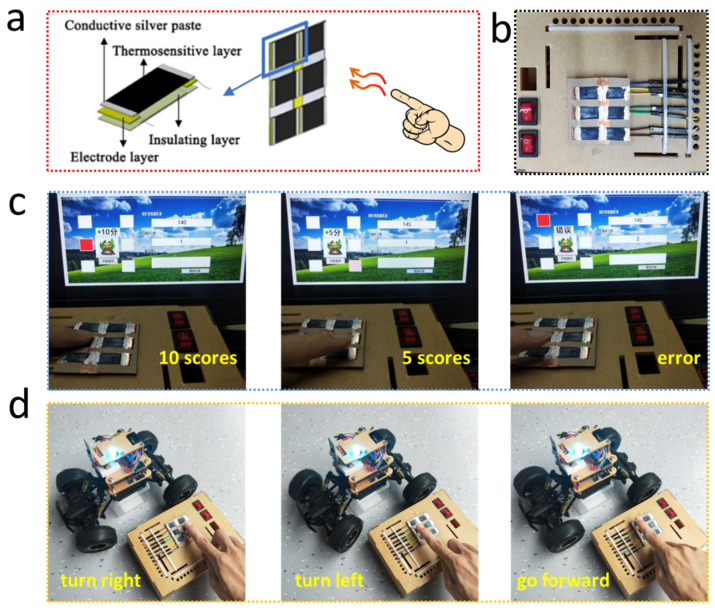
Applications of the flexible PEDOT:PSS temperature-sensitive paper as a noncontact human-machine interface to realize the interaction between player and “Pound-A-Mole” game and a Bluetooth car. (**a**) Schematically illustrating the configuration of the PEDOT:PSS based temperature sensor; (**b**) Optical image of the temperature sensing-based control interface. Application demos of (**c**) “Pound-A-Mole” game and (**d**) Bluetooth car demonstrating the realization of noncontact human-machine control by a temperature sensing-based interface.

The above research shows that the prepared flexible temperature sensor has the advantages of high sensitivity, good flexible, fast response time, and good stability. Combined with the characteristics of simple and large-area preparation, it meets the application requirements of electronic skin, intelligent robot, human-machine interaction interface and other fields. To verify its practical applicability, we integrate the above-mentioned temperature-sensitive paper to a temperature sensing array with 3 × 2 pixels, which acts as a noncontact human-machine interacting interface to control smart vehicle and computer game. The schematic and optical images of the temperature sensing-based control interface are shown in Figure 6a,b, respectively. The designed computer game interface of ground mouse is shown in Figure 6c, which can collect the temperature value of each sensing pixel and responds to it, and the higher the detected proximity temperature, the darker the color of the corresponding square. During gaming, the computer interface will randomly pop out of two different colored squares (light red scores five points and dark red scores ten points). One finger of the player needs to get close to the correct sensing pixel and control the distance between the two to get the correspondence scores. Once the finger accidentally approaches other sensing pixels or touches any sensing pixel, a result “error” is displayed. This game can strengthen the player′s hand control ability and is especially suitable as a fun rehabilitation game for patients with hand dyskinesia. The temperature-sensitive array prepared as shown in Figure 6d can also be used as a noncontact interface to control the movement of the Bluetooth car and realize noncontact human-machine interaction. The six pixels on the sensor array can successfully control the forward, backward, left turn, right turn, and gear shift of the Bluetooth car. In addition to controlling various games, the sensor is also suitable for the control of various public equipment (e.g., elevators and led lights) to reduce the probability of cross-infection during the COVID-19 epidemic. The above experiments show that the flexible temperature-sensitive paper based on PEDOT:PSS has high sensitivity and good stability to detect nearby temperature changes and has promising prospects in artificial intelligence interactive applications in “noncontact” scenarios.

## 5. Conclusions

In summary, we fabricated a noncontact flexible temperature sensor via a simple dip-drying progress, combining the sensitive temperature-responsive property of the PEDOT:PSS with the good hygroscopicity of the printer paper, the temperature-sensitive paper show a good sensing performance including high sensitivity, quick response and good stability within the temperature range of 20–50 °C. To prove the practicability of the noncontact temperature sensor, a 3 × 2 sensing array was prepared as a noncontact human-machine interface to realize the interaction between player and “Pound-A-Mole game” and a Bluetooth car. These two demos show the sensor′s ability to perceive changes in temperature, verifying its application potential as a noncontact human-machine interaction interface.

## Data Availability

Not applicable.
